# Synthesis by Ring-Closing Metathesis and Cytotoxic Evaluation of Novel Thienylmacrolactones

**DOI:** 10.3797/scipharm.1109-09

**Published:** 2011-11-01

**Authors:** Jürgen Krauss, Daniela Köbler, Verena Miller, Franz Bracher

**Affiliations:** Department of Pharmacy – Center for Drug Research, Ludwig-Maximilians University, Butenandtstr. 5–13, 81377 Munich, Germany

**Keywords:** Ring-closing metathesis, Grignard reaction, Macrolactones, MTT assay, Thiophenes

## Abstract

This paper describes the synthesis and biological evaluation of macrolactones containing a thienyl substituent as simple analogues of epothilones. The compounds were prepared in a brief and efficient manner from thiophene-2-carbaldehyde using a ring-closing metathesis with Grubbs I or Grubbs II catalyst as the key step. The target lactones showed only insignificant cytotoxicity, while an intermediate simple thienyl carbinol showed very promising cytotoxicity.

## Introduction

The macrolactone scaffold is found in numerous natural products [1, 2] which show a wide range of pharmacological properties including antimicrobial and cytotoxic activities. Prominent examples are the 14-membered lactone antibiotics erythromycin and telithromycin, as well as their 15-membered, semisynthetic derivative azithromycin [3], the cytotoxic salicylihalamides (12-membered lactones) [4, 5], the 18-membered cytotoxic antibiotic FD-891 [6] and the epothilones, cytotoxic 16-membered lactones which inhibit microtubule function [7]. Extensive investigations on total syntheses [8] and analysis of structure-activity relationships of epothilones have been performed in the past, and a number of substances from this class have entered clinical studies [9]. Sagopilone, a new clinical candidate from the epothilone family [10], demonstrates that the rather complex methylthiazolylpropenyl residue of native epothilones can be replaced by a simple heteroaromatic residue, in this case a benzothiazole.

This prompted us to investigate a new type of 16-membered lactones bearing a sulphur-containing hetarene, namely a thiophene ring [11], next to the lactone oxygen in analogy to new synthetic epothilones. The target compounds were to be prepared using the ring-closing metathesis methodology we worked out earlier for various furyl macrolactones [12, 13].

## Results and Discussion

Thiophene-2-carbaldehyde (**1**) was reacted in a Grignard reaction with pent-4-enyl-magnesium bromide to give alcohol **2**, following a methodology we had worked out earlier for furan derivatives [14, 15]. Secondary alcohol **2** was esterified with undec-10-enoyl chloride to give the ester **3**. From this intermediate containing two terminal vinyl groups the macrolactone **4** was prepared in a ring-closing metathesis under high dilution conditions using either Grubbs I catalyst, benzylidene(dichloro)bis(tricyclohexylphosphane)ruthenium, or Grubbs II catalyst, benzylidene[1,3-bis(2,4,6-trimethylphenyl) 2-imidazolidinylidene]-dichloro(tricyclohexylphosphane)ruthenium. When using Grubbs II catalyst, the reaction proceeded faster than with Grubbs I catalyst (6 h vs. 24 h), for comparable observations see ref. [12, 16, 17].

**Fig. 2:**
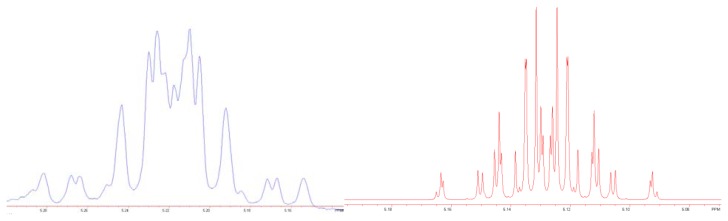
^1^H-NMR resonances of the olefinic protons of the *E*-configurated product 4 (left), and simulated ^1^H-NMR resonances for the *E*-isomer with ^3^*J* = 15.3 Hz for the coupling of the olefinic protons and ^3^*J* = 7.0 Hz for the coupling of the olefinic protons with the neighboring methylene protons.

Both 1^st^ and 2^nd^ generation Grubbs catalysts gave the expected 16-membered lactone **4** as *E*/*Z* mixtures (Grubbs I catalyst: *E*/*Z* = 66:34; Grubbs II catalyst: *E*/*Z =* 77:23). The *E*/*Z* isomers could not be separated by flash column chromatography. The ratio of the isomers was determined by GLC-MS and NMR spectroscopy. The predominating *E*-isomer was identified by the vicinal coupling constant of both olefinic protons of ^3^*J* = 15.3 Hz. Since both olefinic hydrogens showed the same chemical shift, the signal and the coupling constants were verified by computer-aided techniques (NutsPro – NMR Utility Transform Software – Professional, 2D Professional Version – 20020107). The simulated ^1^H-NMR signal and the simulated coupling constants for the olefinic protons of the *E*-isomer were in full accordance to the measured signal and coupling constants (Fig. 2).

Since in preliminary screenings lactone **4** showed negligible cytotoxicity, we intended to introduce hydroxy groups into the ring system to enhance structural similarity to the epothilones. The olefinic double bond of the *E*/*Z* mixture **4** could be dihydroxylated using a *Sharpless* dihydroxylation with β-AD-mix^®^ (OsO_4_, K_3_Fe(CN)_6_, K_2_CO_3_, (DHQD)_2_-PHAL) [14, 18] to give an inseparable mixture of the isomeric diols **5**.

The resulting lactones as well as the precursors **2** and **3** were tested in an agar diffusion assay against several bacteria and fungi. The compounds showed no significant antimicrobial activities compared to tetracycline or clotrimazol.

The cytotoxicity was determined in a MTT assay on HL-60 cells (human leukaemia cell line) using the method of Mosmann [19]. The macrolactones **4** and **5** exhibited only extremely weak cytotoxic activity. Only the secondary alcohol **2** showed high cytotoxic activity in the low μM range (Tab. 1). Compound **5** was also tested in the NCI single high dose cell panel assays on 59 cell lines, but showed no remarkable selectivity or cytotoxicity in this assay.

## Conclusion

In conclusion, we worked out a short and efficient synthesis of novel thienyl macrolactones related to the epothilones. The resulting lactones **4** and **5** did not show significant anticancer or antimicrobial activities.

We supposed that a reason for the negligible cytotoxic activity of lactone **4** might be its very high lipophilicity, as shown by the calculated log P value (Tab. 1). The log P of 7.2 is much higher than the log P limits defined by Lipinski (−0.4 to +5.6) [20] for drug likeness. In fact, the diol **5** (calculated log P = 4.2) showed increased cytotoxicity compared to **4**, but still is far away from being an interesting lead. To our great surprise, the secondary thienyl carbinol **2** showed very interesting cytotoxic activity (IC_50_ = 6 μM). This reminds us of an accidental observation in previous work [12] where a side-product containing a furyl carbinol showed comparable cytotoxicity.

Work is in progress to gain deeper insight into structure-activity relationships of the interesting class of heteroaryl carbinols and to identify their molecular mode of action.

## Experimental

### General

Elemental analysis: Heraeus CHN–Rapid; IR-Spectra: Perkin-Elmer FT-IR Paragon 1000; MS: Hewlett Packard MS-Engine; electron ionisation (EI) 70 eV, chemical ionisation (CI) with CH_4_ (300 eV); NMR: Jeol GSX 400 (^1^H: 400 MHz, ^13^C: 100 MHz); melting points: Büchi Melting Point B-540 (not corrected); flash column chromatography (FCC): silica gel 60 (230–400 mesh, E. Merck, Darmstadt); GLC-MS: Shimadzu GC-17 A (carrier: He, oven temperature program: 100–280 °C, 10 °C/min, capillary column: Varian VF-5ms 30 m × 0.25, split injector T = 250 °C, detector T = 260 °C).

#### (±)-1-(Thiophen-2-yl)-hex-5-en-1-ol (**2**)

To a mixture of 1.53 g (66.5 mmol) magnesium turnings and an iodine crystal in dry THF (25 mL) was added 6.60 g (44.3 mmol) 1-bromopent-4-ene in two portions. The mixture was heated until the Grignard reaction started. The suspension was heated under reflux for further 1 h.

To a solution of 3.30 g (29.5 mmol) thiophene-2-carbaldehyde (**1**) in 15 ml dry THF the prepared solution of pent-4-enylmagnesium bromide in THF was added dropwise. The mixture was stirred for 4 h, than quenched with 30 mL 5 % aqueous NH_4_Cl solution and extracted with diethyl ether (3 × 30 mL). The combined organic layers were dried over Na_2_SO_4_ and the solvent was evaporated. The residue was purified by FCC (n-hexane/ethyl acetate 5:1) to give 4.30 g (80%) of **2** as a colourless oil. ^1^H-NMR (400 MHz, CDCl_3_): δ (ppm) = 1.42 (m, 1H, 3-H), 1.55 (m, 1H, 3-H), 1.85 (m, 2H, 2-H), 2.09 (m, 2H, 4-H), 2.16 (s, 1H, OH), 4.90 (t, *J* = 6.5 Hz, 1H, 1-H), 4.95 (m, 1H, 6-H), 5.01 (m, 1H, 6-H), 5.79 (m, 1H, 5-H), 6.95 (m, 2H, 2′-H, 3′-H), 7.23 (m, 1H, 4′-H). ^13^C-NMR (400 MHz, CDCl_3_): δ (ppm) = 25.0 (C-3), 33.4 (C-4), 38.7 (C-2), 70.2 (C-1), 114.8 (C-6), 123.7 (C-5′), 124.5 (C-3′), 126.6 (C-4′), 138.4 (C-5), 148.8 (C-2′). EI-MS m/z (rel. int.): 182 (M^+^, 100), 164 (M^+^-18, 30), 97 (75). IR (NaCl, film): ν [cm^−1^] = 3379, 3074, 2936, 1860, 1640, 1439, 1414, 1066, 1029, 995, 912, 852, 830, 699. Elemental analysis: C_10_H_14_OS (182.29). Calcd.: C: 65.89. H: 7.74. S: 17.59. Found: C: 65.71. H: 7.91. S: 17.17.

#### (±)-1-(Thiophen-2-yl)hex-5-en-1-yl undec-10-enoate (**3**)

1.60 g (8.80 mmol) of **2** were dissolved in 30 mL dry toluene, 2.10 g (10.6 mmol) undec-10-enoyl chloride and 5 mL *N*-ethyl-*N*,*N*-dimethylamine (EDMA) were added. The mixture was stirred for 5 h and then the solvent was evaporated. The residue was dispersed in 20 mL water and extracted with diethyl ether (3 × 30 mL). The combined organic layers were dried over Na_2_SO_4_ and the solvent was evaporated. The residue was purified by FCC (n-hexane/ethyl acetate 10:1 to 5:1) to give 1.85 g (60%) of **3** as a colourless oil. ^1^H-NMR (400 MHz, CDCl_3_): δ (ppm) = 1.26 (m, 8H, 4 CH_2_), 1.36 (m, 4H, 2 CH_2_), 1.58 (m, 2H, CH_2_), 1.89 (m, 1H, CH_2_), 2.03 (m, 3H, CH_2_, CH_2_), 2.09 (m, 2H, CH_2_), 2.30 (t, *J* = 8.0 Hz, 2H, CH_2_), 4.96 (m, 4H, 2 =CH_2_), 5.79 (m, 2H, 2 -CH=), 6.04 (t, *J* = 7.4 Hz, 1H, CH), 6.95 (dd, *J* = 4.9 Hz, *J =* 3.4 Hz, 1 H, aromat. CH), 7.03 (d, *J* = 3.4 Hz, 1H, aromat. CH), 7.25 (dd, *J* = 4.9 Hz, *J* = 1.1 Hz, 1H, aromat. CH). ^13^C-NMR (400 MHz, CDCl_3_): δ (ppm) = 24.8 (CH_2_), 24.9 (CH_2_), 28.9 (CH_2_), 29.0 (CH_2_), 33.2 (CH_2_), 33.8 (CH_2_), 34.5 (CH_2_), 35.8 (CH_2_), 70.8 (CH), 114.1 (=CH_2_), 115.0 (=CH_2_), 125.1 (aromat. CH-), 125.7 (aromat. CH-), 126.5 (aromat. CH-), 138.2 (=CH-), 139.2 (=CH-), 143.7 (quart. C), 173.1 (CO). EI-MS m/z (rel. int.):348 (M^+^, 2), 182 (100), 97 (94). Elemental analysis: C_21_H_32_O_2_S (348.55). Calcd.: C: 72.37. H: 9.25. S: 9.20. Found: C: 72.99. H: 9.78. S: 8.60.

#### (±)-16-(Thiophen-2-yl)-oxacyclohexadec-11-en-2-one (**4**) (E/Z-mixture)

1.0 g (2.9 mmol) of **3** and 0.16 g (0.20 mmol) of Grubbs I catalyst (or 0.17 g (0.20 mmol) Grubbs II catalyst) were dissolved separately in 5 mL dry toluene each. Using two syringe pumps these solutions were simultaneously added dropwise to 250 mL of boiling dry toluene over a period of 12 h for Grubbs I and of 4 h for Grubbs II catalyst. The mixture was heated under reflux for another 12 hours under a N_2_-atmosphere for Grubbs I and 2 h for Grubbs II catalyst. Then the solvent was evaporated and the residue was purified by FCC (n-hexane/ethyl acetate 9:1) to give 690 mg (74% for Grubbs I), 790 mg (86 % for Grubbs II) of **4** as a colourless oil. ^1^H-NMR (400 MHz, D_6_-benzene): resonances of the predominating *E*-isomer: δ (ppm) = 1.28 (m, 10H, 5 CH_2_), 1.55 (m, 4H, 2 CH_2_), 1.94 (m, 4H, 2 CH_2_), 2.25 (m, 4H, 2 CH_2_), 5.22 (m, 2H, 2 CH= containing a coupling constant of *J* = 15.3 Hz), 6.32 (t, *J* = 7.8 Hz, 1H, CH), 6.90 (dd, *J* = 4.9 Hz, *J =* 3.2 Hz, 1H, aromat. CH), 6.99 (d, *J* = 3.2 Hz, 1H, aromat. CH), 7.20 (m, 1H, aromat. CH). ^13^C-NMR (400 MHz, D_6_-benzene): resonances of the predominating *E*-isomer: δ (ppm) = 25.2 (CH_2_), 25.6 (CH_2_), 26.6 (CH_2_), 27.4 (CH_2_), 28.1 (CH_2_), 28.2 (CH_2_), 28.3 (CH_2_), 32.0 (CH_2_), 32.2 (CH_2_), 34.9 (CH_2_), 35.7 (CH_2_), 71.0 (CH), 125.4 (aromat. CH), 126.0 (aromat. CH), 126.5 (aromat. CH), 130.5 (-CH=), 132.0 (-CH=), 143.3 (quart. C), 173.1 (CO). CI-MS m/z (rel. int.): 320 (M^+^, 15), 110 (100). HR-MS: Calcd.: 320.1810. Found.: 320.1810. Elemental analysis: C_19_H_28_O_2_S (320.50). Calcd.: C: 71.28. H: 8.81 S: 10.00. Found: C: 71.87. H: 9.08. S: 9.90.

#### (±)-11,12-Dihydroxy-16-(thiophen-2-yl)-oxacyclohexadecan-2-one (**5**) (mixture of stereoisomers)

250 mg (0.781 mmol) of lactone **4** were dissolved in 50 mL *tert.*-butanol/water (1:1) and 5.5 g β-AD mix^®^ (Aldrich) were added. The suspension was stirred for 12 h at room temperature. Then it was quenched with 100 mL 5 % aqueous Na_2_S_2_O_3_ solution and extracted with diethyl ether (3 × 30 mL). The combined organic layers were dried over Na_2_SO_4_, the solvent was evaporated and the residue was purified by FCC (n-hexane/ethyl acetate 1:1) to give 210 mg (76%) of **5** as a colourless oil. ^1^H-NMR (400 MHz, CDCl_3_): δ (ppm) = 1.31 (m, 14H, 7 CH_2_), 1.63 (m, 4H, 2 CH_2_), 1.99 (m, 2H, CH_2_), 2.34 (t, *J* = 7.2 Hz, 2H, CH_2_), 3.49 (m, 2H, CH), 5.24 (m, 1H, CH), 6.95 (m, 2H, 2 aromat. CH), 7.25 (m, 1H, aromat. CH). ^13^C-NMR (400 MHz, CDCl_3_): δ (ppm) = 23.3–34.0 (11 CH_2_), 73.6 (CH), 75.7 (CH), 81.5 (CH), 123.1 (aromat. CH), 124.4 (aromat. CH), 126.8 (aromat. CH), 146.3 (quart. C), 179.4 (CO). MS (EI): m/z (%) = 354 (M^+^, 10), 336 (5), 326 (15), 168 (27), 126 (100), 97 (65). HR-MS: C_19_H_30_O_4_S (354.51). HR-MS: Calcd.: 354.1865 Found: 354.1865.

## Figures and Tables

**Fig. 1:**
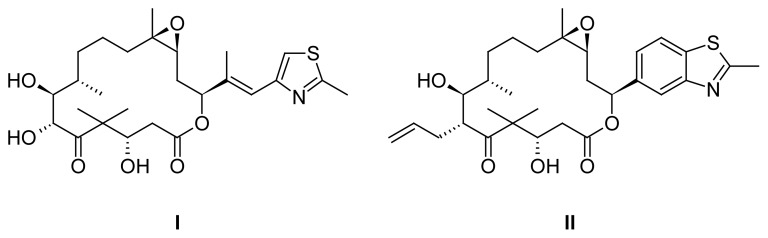
Structures of epothilone B (**I**) and sagopilone (**II**)

**Sch. 1:**
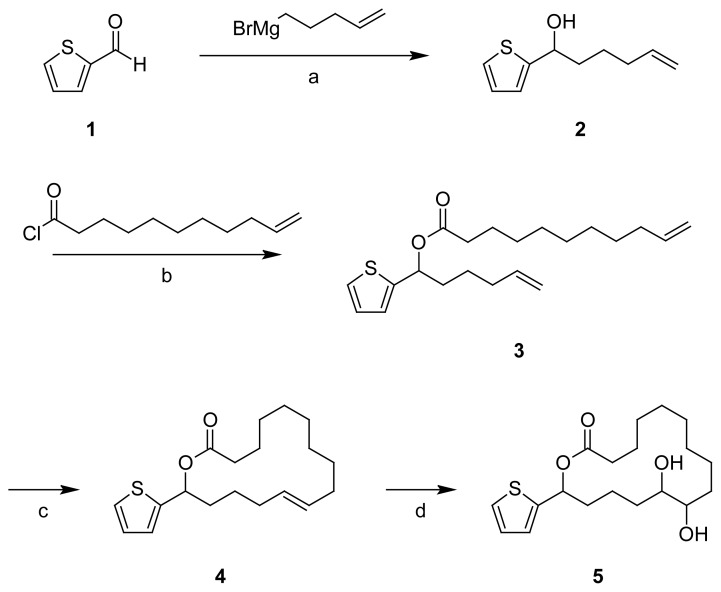
a: THF. b: toluene, EDMA. c: toluene, Grubbs catalyst (1^st^ or 2^nd^ generation), high dilution technique. d: *tert*.-butanol/H_2_O, β-AD mix^®^.

**Tab. 1:** Cytotoxicity against HL 60 cells.

compound	IC_50_ [μM (μg/mL)]	log P (calcd.)
**2**	6 (1.1)	3.2
**3**	> 100	7.2
**4**	290 (92.8)	6.1
**5**	120 (42.5)	4.2
**cisplatin**	5 (1.5)	
